# Lower Levels of Vitamin D Are Associated with an Increase in Insulin Resistance in Obese Brazilian Women

**DOI:** 10.3390/nu13092979

**Published:** 2021-08-27

**Authors:** Minna F. Schleu, Beatriz Barreto-Duarte, Maria B. Arriaga, Mariana Araujo-Pereira, Ana Marice Ladeia, Bruno B. Andrade, Maria L. Lima

**Affiliations:** 1 Programa de Pós-Graduação em Medicina e Saúde Humana, Escola Bahiana de Medicina e Saúde Pública (EBMSP), Salvador 40290-000, Brazil; minna.schleu@gmail.com (M.F.S.); anamarice@bahiana.edu.br (A.M.L.); mlourdeslima@bahiana.edu.br (M.L.L.); 2 Laboratório de Inflamação e Biomarcadores, Instituto Gonçalo Moniz, Fundação Oswaldo Cruz, Salvador 40296-710, Brazil; beatrizbbd@hotmail.com (B.B.-D.); mbag711@gmail.com (M.B.A.); araujopereira.mariana@gmail.com (M.A.-P.); 3 Multinational Organization Network Sponsoring Translational and Epidemiological Research (MONSTER) Initiative, Salvador 41810-710, Brazil; 4 Curso de Medicina, Universidade Salvador (UNIFACS), Laureate Universities, Salvador 41720-200, Brazil; 5 Programa de Pós-Graduação em Clínica Médica, Universidade Federal do Rio de Janeiro, Rio de Janeiro 21941-617, Brazil; 6 Faculdade de Medicina, Universidade Federal da Bahia, Salvador 40110-100, Brazil

**Keywords:** vitamin D, obesity, women, insulin resistance, HOMA-IR

## Abstract

Adult women are more likely to be obese than men. Moreover, there is evidence that obesity is a risk factor for increased insulin resistance (IR) and hypovitaminosis D (VITD), conditions related to metabolic and endocrinologic disturbance. We performed a cross-sectional study with 103 women diagnosed with obesity, recruited between 2009 and 2013, in an obesity referral outpatient clinic in Bahia, Brazil. Laboratory and clinical characteristics were compared between the groups according to the degree of obesity (I, II and III), and levels of 25-hydroxyvitamin D [25(OH)D] were used to define the VITD status (insufficiency and no insufficiency). We calculated the homeostatic model assessment-IR (HOMA-IR) index to assess insulin resistance in the groups. Our analyses revealed that HOMA-IR values and VITD levels were inversely correlated. Furthermore, we observed a distinct expression profile of values of laboratory markers according to 25(OH)D levels. Negative correlations were found between HOMA-IR and body mass index (BMI) in VITD insufficient participants but not in those with the sufficiency. Furthermore, multivariate regression demonstrated independent associations between lower levels of 25(OH)D and increased values of HOMA-IR. These findings suggests that lower levels of VITD are strongly associated with the increased IR in obese women.

## 1. Introduction

Obesity is a worldwide public health problem, with an average prevalence reaching 13% of adult individuals, with women being more affected [1]. In Brazil, according to the Brazilian Institute of Geography and Statistics (Instituto Brasileiro de Geografia e Estatística, IBGE), approximately 30% of Brazilians are obese, with female individuals representing 60% of this population [2]. This disease has been defined by the World Health Organization (WHO) as a body mass index (BMI) equal to or greater than 30 kg/m^2^ [1]. Several studies have linked obesity to the deficiency of essential vitamins and minerals [3], including vitamin D (VITD) [4,5]. VITD deficiency has a direct impact on the production and signaling of insulin pathways which may accelerate and contribute to the establishment of insulin resistance (IR) [6,7]. Importantly, women are often a target of study, since in addition to the risks of obesity observed in the general population [8,9], there is also a negative impact of VITD deficiency on both fertility and contraception [10]. Recent studies have shown that VITD has an important role in the risk of glucose intolerance [11,12,13]. This vitamin exhibits a hypoglycemic function, which is justified by stimulating the expression of insulin receptors on the cell membrane and through increasing insulin secretion by pancreatic cells. Whether by improving the response or increasing insulin secretion, the association between low VITD levels and dysglycemia is the goal of studies aiming at a better understanding of the pathogenesis of diabetes, with supplementation of this vitamin being considered as a potential adjunctive therapy.

Furthermore, due to the fat-soluble characteristic of VITD, in obese patients, 25hydroxyvitamin D (25(OH)D) is sequestered by the abundant adipose tissue resulting in low levels of VITD in the peripheral blood of this population [14,15,16]. This makes obesity a risk factor for hypovitaminosis D and a possible risk factor for insulin resistance. In the present study, we investigated the presence of VITD deficiency in obese women as a risk factor for increased insulin resistance through an evaluation of laboratory, clinical and epidemiological variables of this population.

## 2. Materials and Methods

### 2.1. Study Design and Participants

In this cross-sectional study, performed between 2009 and 2013, blood samples were collected from 103 women diagnosed with obesity, enrolled and followed up at the obesity clinic in the city of Salvador in the state of Bahia. The aforementioned institution is a referral center in the care and treatment of obesity diseases in the state of Bahia, Brazil.

We only use baseline data from patients who entered the specialized service. Comparisons within the obese group were performed between participants stratified based on use of BMI considering: Obesity I: BMI = 30.0–34.9 kg/m^2^; Obesity II: BMI = 35.0–39.9 kg/m^2^; Obesity III: BMI ≥ 40.0 kg/m^2^ [17]. In addition, comparisons were performed stratifying the participants according with the level of 25(OH)D: participants were considered VITD deficient (25(OH)D level: >20 or ≤30 ng/mL), insufficient (25(OH)D level: <20 ng/mL) and normal (25(OH)D level: >30 ng/mL). Additionally, we compared the status of 25(OH)D as insufficient (25(OH)D level < 20 ng/mL) and not insufficient, which includes insufficient and normal levels (25(OH)D level > 20 mg/mL) [18]. Participants included in the study were also interviewed by filling out a questionnaire including data such as age, ethnicity (self-reported), diabetes, hypertension, use of obesity medication and smoking. Some patients were diagnosed as diabetics or hypertensive with the measurements of the reference center. The anthropometric measurements obtained were weight, height, BMI and waist circumference. The analyses were performed stratifying the status of VITD as insufficient and not insufficient.

#### Inclusion and Exclusion Criteria

Women with BMI ≥ 30 kg/m^2^ over 18 years were included. Patients receiving current supplementation or in the last 3 months with calcium and vitamin D, such as, patients with chronic renal failure and pregnancy, were excluded from the analyses.

### 2.2. Laboratory Measurements

Blood samples were obtained after a fasting period of at least 12 h. The following parameters were assessed in a reference laboratory: C-reactive protein (CRP), thyroid stimulating hormone (TSH), fasting plasma glucose (FPG) (mg/dL), glycated hemoglobin (HbA1C) (%), fasting insulin with HOMA-IR calculation, creatinine(mg/dL), blood urea nitrogen (BUN) (mg/dL), serum calcium (mg/dL), albumin (mg/dL) and 25(OHD)(ng/mL). The measurements of triglycerides, total cholesterol and high-density lipoprotein cholesterol (HDL) were performed according to the specifications of the manufacturers. The Friedewald formula was used to calculate low-density lipoprotein cholesterol (LDL) from total cholesterol, triglycerides and HDL. ELISA commercial kits from R&D Systems (Minneapolis, MN, USA) were used to quantify concentrations of 25(OH)D.

### 2.3. Statistical Analysis

Descriptive statistics were performed to characterize the study population. Kolmogorov–Smirnoff test was used to evaluate the Gaussian distribution of the quantitative variables. Categorical variables were presented as frequency and proportions and compared using a two-sided Pearson’s chi-squared test or Fisher’s two-tailed test in 2 × 3 or 2 × 2 tables, respectively. Quantitative variables were presented as median and interquartile range (IQR) values and compared using the Mann–Whitney *U* (between 2 groups) or Kruskal–Wallis test (between >2 groups).

Hierarchical cluster analyses (Ward’s method) of log10 transformed values and z-score normalized data were employed to depict the overall expression profile of biomarkers in the study subgroups. Correlations between the different parameters were evaluated with the Spearman test. A linear regression model was performed to evaluate the independent associations between clinical and biochemical characteristics of the patients and the increased HOMA-IR. Only relevant parameters in the literature were included in the multivariable model; *p*-values of <0.05 were considered statistically significant.

## 3. Results

### 3.1. Characteristics of the Study Population

Between 2009 and 2013, a total of 103 women with diagnosis of obesity were enrolled. First, we stratified the cohort according to the obesity class: 32 with obesity I, 40 with obesity II and 31 with obesity III. In this population, pardo (42.6%) and black (43.6%) were the predominant racial groups, and the median age was 44 years (IQR = 34–54 years, *p* = 0.193). Interestingly, age, race, life habits and comorbidities such as alcohol use, smoking, hypertension and diabetes were not statistically significant in the comparison between groups, as well as the use of drugs for obesity (Table S1). Next, the patients were grouped according to 25(OH)D status: 76 with no insufficiency levels and 27 with insufficiency. The groups had similar clinical and laboratory characteristics, except for the variable HOMA-IR (*p* = 0.018), presenting higher levels in the insufficiency group (median: 4.99; IQR: 3.49–5.91) in contrast to no insufficiency (3.77; IQR: 2.77–5.1) (Table 1).

### 3.2. Laboratory Evaluation between the Clinical Groups

Laboratory information was assessed and compared between the groups (Table 1). The lipid profile of the study patients was similar, but, in contrast, the values of thyroid-stimulating hormone (TSH) (*p* = 0.024) and C-reactive protein (CRP) (*p* = 0.040) were significantly different between the study groups. Of note, TSH and CRP levels were higher in patients with obesity III. These findings suggest a more pronounced proinflammatory status in patients with a more severe degree of obesity.

The levels of FPG and HbA1c were similar between groups, whereas the values of HOMA-IR (*p* = 0.031) and insulin (*p* = 0.006) were different, so that the group of patients with obesity III exhibited the highest HOMA-IR value, and the group of patients with obesity II had the highest insulin value. Of note, the level (*p* = 0.891) and status of 25(OH)D (*p* = 0.531) were similar in the study population.

### 3.3. Lower Levels of 25 (OH)D Were Associated with Higher Insulin Resistance in Obese Women

**Figure 1:**
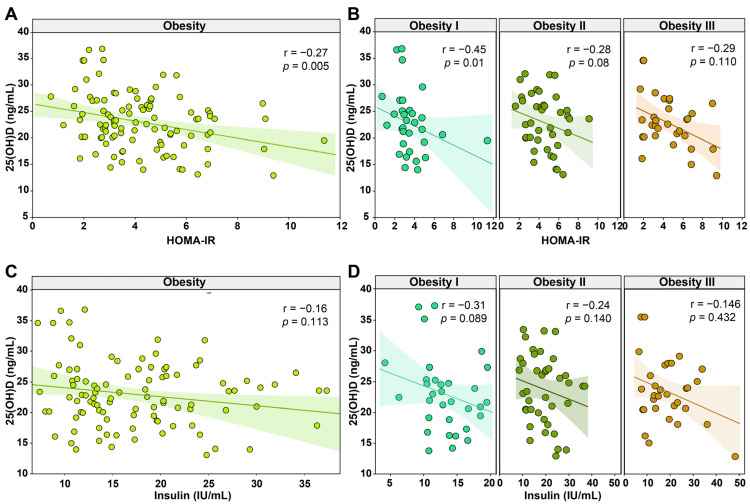
Spearman correlation analysis of the 25-hydroxyvitamin D [25(OH)D] shows association with HOMA-IR and insulin in women with obesity. (**A**) Correlation plot between 25-hydroxyvitamin D and HOMA-IR. (**B**) Correlation plot between 25-hydroxyvitamin D and HOMA-IR by obesity class. (**C**) Correlation plot between 25-hydroxyvitamin D and Insulin. (**D**) Correlation plot between 25-hydroxyvitamin D and insulin by obesity class. Abbreviations: 25(OH)D = 25-hydroxyvitamin D; HOMA-IR = homeostatic model assessment- insulin resistance; IU = international unit; mL = microliter.

Given the results reported above, we further investigated the possible impact of the levels of 25(OH)D on insulin resistance. We performed the Spearman correlation analysis to directly evaluate the associations between the levels of 25(OH)D and the resistance of insulin according to the HOMA-IR and with the insulin levels. We found that the level of 25(OH)D was negatively associated with HOMA-IR values (r = −0.27; *p* = 0.005), being the only parameter that showed statistical significance (Figure 1A). Correlations between 25(OH)D levels and the other parameters are shown in Figure S1. Interestingly, in the associations stratified by class of obesity, we found a negative tendency in the correlation between low levels of 25(OH)D in all groups, but only obesity I presented statistical significance (r = −0.45; *p* = 0.01) (Figure 1B). The associations between insulin levels and 25(OH)D levels were not statistically significant in the general population and stratifications by the class of obesity, but we also found a negative tendency (Figure 1C,D). These findings highlight mechanisms of peripheral insulin resistance in obese women that were influenced by the circulating 25(OH)D levels and the class of obesity.

**Figure 2:**
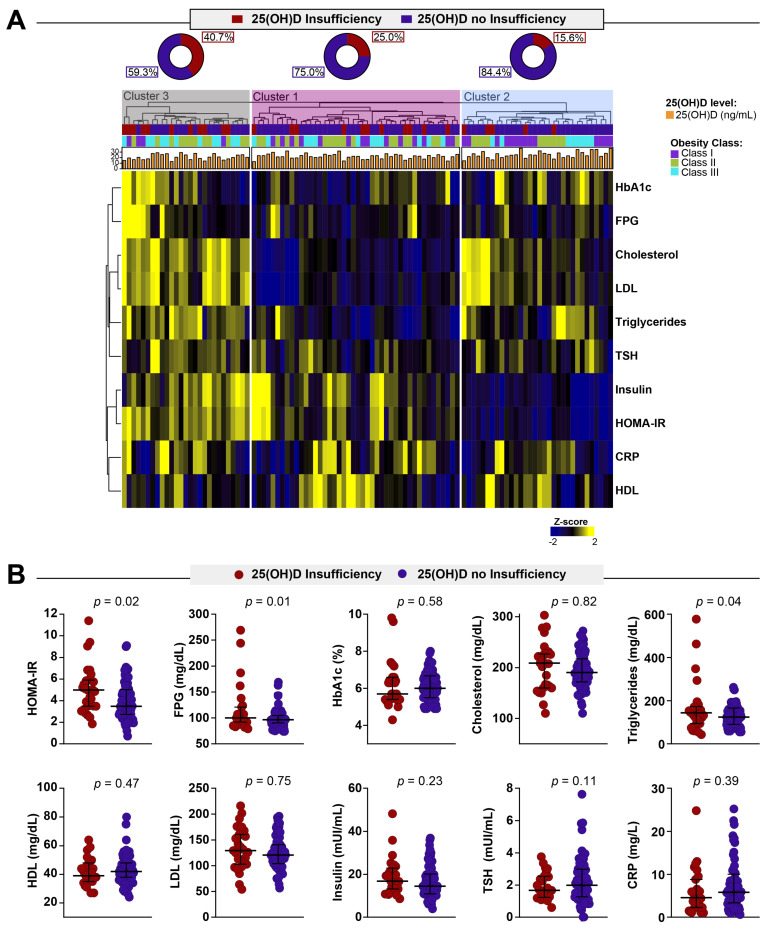
Biochemical profiles of obese women stratified by 25-hydroxyvitamin D status. (**A**) Value of each parameter was log10 transformed. Mean values for each indicated clinical group were z-score normalized, and a hierarchical cluster analysis (Ward’s method with 100xbootstrap) was performed to illustrate the overall biochemical profiles. (**B**) Data represent median and interquartile ranges. The Mann–Whitney *U* test was employed to compare the values detected between the study subgroups. Abbreviations: FPG = Fasting plasma glucose; HbA1c = Glycated hemoglobin; HDL: High-density lipoprotein; LDL = Low-density Lipoproteins; TSH = Thyroid-stimulating hormone; C-RP = C-reactive protein; 25(OH)D = 25-hydroxyvitamin D.

Corroborating the idea that insulin resistance is associated with insufficient levels of VITD, we extend our analysis to assess a laboratorial profile of the groups. We employed a hierarchical cluster analysis using laboratory parameters measured in peripheral blood to identify a profile of the obese women presenting with different status of VITD (Figure 2A). This approach revealed that patients with lower levels of VITD and obesity class II or III tend to present a distinct profile hallmarked by higher values of HbA1c, FPG, Cholesterol, LDL, triglycerides, TSH, insulin and HOMA-IR. Of note, we also identified that the group with a distinct profile hallmarked by higher values of laboratory parameters was mostly composed of patients with obesity type II and type III. We next described in detail the associations of biochemical parameters with 25(OH)D status (Figure 2B). We found that the median values of HOMA-IR, FPG and triglycerides were significantly higher in obese women with 25(OH)D insufficiency (Figure 2B). These findings corroborate the hypothesis that a higher degree of obesity as well as VITD insufficiency may predispose one to immunologic and metabolic disturbance.

### 3.4. VITD Insufficiency Was Independently Associated with an Increase in HOMA-IR

**Figure 3:**
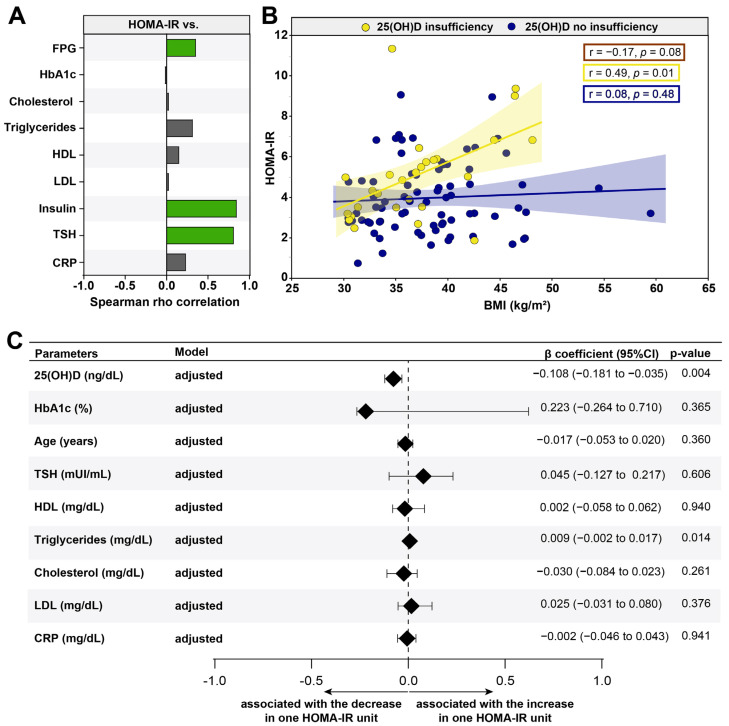
Factors associated with HOMA-IR among women with obesity. (**A**) Spearman rho correlation between HOMA-IR and clinical and biochemical parameters; statistically significant differences are highlighted in green (positive) and yellow (negative). (**B**) Spearman correlation between HOMA-IR and BMI levels in women with obesity grouped according to 25-hydroxyvitamin D status; the values of r and p shown in the figure correspond to the general population (red box), with insufficient VID D (yellow box) and without insufficient VITD (blue box). (**C**) A linear regression analysis was used to test independent association between clinical and biochemical parameters and HOMA-IR. Abbreviations: FPG = Fasting plasma glucose; HbA1c = Glycated hemoglobin; HDL: High-density Lipoprotein; LDL = Low-density Lipoproteins; TSH = Thyroid-stimulating hormone; CRP = C-reactive protein; 25(OH)D = 25-hydroxyvitamin D.

Furthermore, we described which laboratory exams had relationships with HOMA-IR and contributed to insulin resistance. As expected, FPG (r = 0.20; *p* = 0.047) and insulin (r = 0.85; *p* < 0.001) values were directly correlated with elevations in HOMA-IR values (Figure 3A). Interestingly, the levels of TSH (r = 0.81; *p* = 0.024) were associated with an increase in peripheral insulin resistance (Figure 3A). We also evaluated the relationships between 25(OH)D sufficiency and insufficiency according to the HOMA-IR and BMI in the patients (Figure 3B). We observed that gradual increases in HOMA-IR values were related to remarkable increases in values of BMI in obese women with 25(OH)D insufficiency (r = 0.49; *p* = 0.01); the same behavior was not observed in the population in general (r = −0.17; *p* = 0.08) as well as in the obese women without 25(OH)D insufficiency (r = 0.08; *p* = 0.48) (Figure 3B). These findings indicate that the VITD insufficiency was directly associated with substantial changes in concentrations of laboratorial parameters that relate to an insulin resistance.

Finally, a linear regression analysis was performed to test independent associations between the parameters analyzed and the higher values of the HOMA-IR value (Figure 3C). We found that a decreased 25(OH)D (β coefficient = −0.108; 95%CI: −0.181 to −0.035; *p* = 0.004) was associated with an increase in the HOMA-IR value. Additionally, an increase of one unit in triglyceride levels (β coefficient = 0.009; 95% CI: −0.002 to 0.017; *p* = 0.014) was associated with an increase of one unit in the HOMA-IR, consequently impacting the insulin resistance (Figure 3C). Importantly, the significantly stronger correlation with TSH and HOMA-IR disappears when we apply the adjusted model. These results highlight the importance of 25(OH)D insufficiency as a risk factor in obese women to develop and/or aggravate insulin resistance.

## 4. Discussion

VITD is a metabolite of huge clinical importance due to its interactions with calcium and the bone system in general. In addition, many studies suggest that low levels of VITD are associated with increased frequency of immune disorders and other diseases, such as obesity. Importantly, obesity is a risk factor for hypovitaminosis D and is closely related to the development of other comorbidities: for example, fertility disorders and cardiovascular diseases, especially in women [8,9]. In the present study, we aimed to investigate the association between lower levels of VITD and peripheral IR in a group of obese Brazilian women.

In the first place, the study population was obese and had several comorbidities such as diabetes and hypertension. Interestingly, these comorbidities were not significant between the class of obesity and 25(OH)D status, showing that our population was similar in these aspects regardless of the class of obesity presented. Lifestyle habits such as smoking, alcohol consumption and physical activity showed the same behavior, being similar in populations with different obesity classes and 25(OH)D statuses. These findings are in disagreement with the literature, which links a high class of obesity with poor lifestyle habits and a greater number of comorbidities [19,20]. We hypothesized that this may have occurred due to the small number of patients in each arm of the study, which may have impacted the analysis employed. Of note, insufficient patients have different fasting blood glucose levels than non-insufficient patients, but the same behavior was not noted in HbA1c. We theorize that this behavior may occur because some patients had the diagnosis of diabetes in the baseline visit to the center, and others had the diagnosis before and were using glycemic control medications.

Obesity is a chronic disease that triggers a disturbance in the immune system, leading to a sustained proinflammatory status [21,22]. This pro-inflammatory status has already been described by several authors and is closely related to increased cardiovascular risk and cardiovascular diseases, coagulation disorders, IR, atherosclerosis and DM [20]. Importantly, chronic inflammation is also linked with the development of metabolic syndrome [21], which is a set of signs and symptoms (changes in HDL and triglycerides levels, elevated blood pressure and blood glucose and elevated abdominal circumference) that mark a higher cardiovascular risk and metabolic alterations [23], being related to low levels of VITD [24], and is considered an important factor in increasing the morbidity and mortality of obese patients. We found that the profile of obese women who had insufficient levels of VITD had an increase in proinflammatory markers, exhibiting a district profile compared with the patients who did not present insufficiency. In addition, several studies indicate that obese patients have larger systemic inflammation [21]; therefore, adequate levels of VITD are important due to their modulating and regulatory function of the immune system. Our findings are in agreement with the literature, since patients with higher degrees of obesity and lower levels of VITD present a pro-inflammatory profile exacerbated with CRP levels and, in addition to lipid profile and HOMA-IR, show a superior metabolic risk and persistent immune activation. Of note, we also observed an increase in triglyceride levels with insufficient levels of VITD. This fact could be justified due to the absence of the action of VITD metabolites that increase the expression of lipoprotein lipase [25,26] in vitro [27], as well as the increased number of patients with peripheral insulin resistance in this group, which may cause a decrease in triglyceride levels.

Obesity is a risk factor for peripheral IR; moreover, patients with lower levels of VITD had a higher value of HOMA-IR. Several studies report that VITD has receptors that are located in numerous cells of the body, such as adipocytes, influencing the expression of adipogenic genes and apoptosis [25,28,29]. In accordance with our findings, a double-blind randomized clinical trial showed that the administration of high doses of VITD in obese patients with peripheral insulin resistance or pre-DM increased the sensitivity to insulin and reduced the chance of progression of the condition to DM [30].

Another point worth noting in our study was that the increase in BMI showed to be inversely correlated with the increase in peripheral insulin resistance in obese women with insufficient levels of 25 (OH)D, but not in the general population or in patients 25(OH)D non-insufficiency. Interestingly, similar findings have been reported in large cross-sectional studies in which VITD levels are inversely correlated with BMI [31,32]. Additionally, observational studies have found a strong association between low levels of VITD and pre-DM [7], obesity or metabolic syndrome [24], suggesting a significant metabolic alteration associated with low levels of VITD. Of note, we also found that the VITD and triglyceride levels were independently associated with the increase in peripheral insulin resistance that was previously described as an independent relationship, in which high triglyceride levels were associated with increased peripheral insulin resistance and reduced function of insulin pancreatic beta cells.

This study has limitations. The sample size is small due to the difficulty in obtaining the necessary laboratory parameters in suitable patients for the study, and the cross-sectional study design does not allow us to establish a causal effect between the study findings. In addition, we do not have a control group, which increases the chances of possible interferences in the findings. Furthermore, HOMA-IR is not the gold standard for measuring insulin resistance. Therefore, further studies with larger sample groups and patient follow-up are needed so that we can better understand the dynamics established between 25(OH)D levels and peripheral insulin resistance in obese patients. Despite the limitations, our study provides consistent evidence that low levels of 25(OH)D are associated with an increase in peripheral insulin resistance. We also observed that the laboratory profile of patients differs according to the sufficiency or insufficiency of VITD; we hypothesize that this profile is justified by a greater immunological disturbance, and further investigations are necessary to better understand the relationship between VITD, obesity, peripheral insulin resistance and immunity.

## Figures and Tables

**Table 1:** Clinical and biochemical characteristics of the population by 25(OH)D status.

Characteristics	No Insufficiency	Insufficiency	*p*-Value
	*n* = 76	*n* = 27	
Age (years)—median (IQR)	44 (33–53.5)	42 (37–54)	0.784
Race—*n* (%)			0.478
White	11 (15.94)	2 (8)	
Pardo	27 (39.13)	13 (52)	
Black	31 (44.93)	10 (40)	
Waist circumference—median (IQR)	110 (104–116)	108 (103–114)	0.363
BMI (kg/m^2^)-median (IQR)	37.755 (33.74–41.16)	36.98 (32.8–39)	0.802
Obesity Class-n (%)			0.547
Class I	22 (28.95)	10 (37.04)	
Class II	29 (38.16)	11 (40.74)	
Class III	25 (32.89)	6 (22.22)	
Smoking (current)-n (%)	2 (2.67)	0 (0)	1.000
Smoking (past)-n (%)	14 (19.44)	5 (18.52)	1.000
Passive smoking-n (%)	10 (14.08)	3 (11.11)	1.000
Alcohol consumption (current)-n (%)	23 (31.51)	10 (37.04)	0.777
Alcohol consumption (past)-n (%)	12 (17.39)	4 (18.18)	1.000
Use of obesity medication-n (%)	13 (52.0%)	4 (57.1%)	1.000
Physical Activity-n (%)	19 (26.0%)	8 (29.6%)	0.915
Hypertension-n (%)	41 (54.67)	18 (66.67)	0.392
Diabetes-n (%)	13 (17.11)	9 (33.33)	0.135
Biochemistry parameters			
Cholesterol (mg/dL)-median (IQR)	190.5 (172–216)	209 (160–227)	0.429
Triglycerides (mg/dL)-median (IQR)	124 (90–166)	134 (94–173)	0.534
LDL (mg/dL)-median (IQR)	122.4 (104.4–141.2)	126.2 (103–161)	0.372
HDL (mg/dL)-median (IQR)	42 (38–48)	39 (35–48)	0.380
TSH (mUI/mL)-median (IQR)	1.99 (1.28–2.98)	1.67 (1.27–2.51)	0.424
CRP (mg/L)-median (IQR)	5.05 (2.87–8.98)	4.57 (2.26–11)	0.672
FPG (mg/dL)-median (IQR)	96 (92–106)	94 (85–108)	0.896
HbA1c (%)-median (IQR)	5.9 (5.5–6.6)	5.7 (5.4–6.4)	0.663
Insulin (mUI/mL)-median (IQR)	14.45 (10.9–20.2)	16.8 (13.2–22.9)	0.254
HOMA-IR-median (IQR)	3.77 (2.77–5.1)	4.99 (3.49–5.91)	0.018

Data represent no. (%), except for age and BMI, which are presented as median and interquartile range (IQR). All: Obesity was classified in class I: BMI 30.0–34.9 kg/m^2^; II: BMI = 35.0–39.9 kg/m^2^; and III: BMI = ≥ 40.0 kg/m^2^. Physical activity was defined as at least 30 min of activity at least 3 times a week. Continuous variables were compared between obesity class using Kruskal–Wallis test. Diabetes and hypertension were self-reported and diagnosed. Abbreviations: BMI = Body mass index; LDH = Low-density lipoproteins; HDL: High-density lipoprotein; TSH = Thyroid-stimulating hormone; CRP = C-reactive protein; FPG = Fasting plasma glucose; HbA1c = Glycated hemoglobin; 25(OH)D = 25-hydroxyvitamin D.

## Data Availability

Data will be available upon request.

## Supplementary Materials

The following are available online at https://www.mdpi.com/article/10.3390/nu13092979/s1, Table S1. Clinical and biochemical characteristics of the population by obesity type. Figure S1. Spearman correlation analysis of the 25-hydroxyvitamin D [25(OH)D] and biochemical parameters in women with obesity. Correlation plot between 25-hydroxyvitamin D and all the biochemical parameters evaluated in the study.

## Abbreviations

FPG	Fasting Plasma Glucose
HbA1c	Glycated Hemoglobin
HDL	High-Density Lipoprotein
LDL	Low-Density Lipoproteins
TSH	Thyroid-Stimulating Hormone
C-RP	C-Reactive Protein
25(OH)D	25-hydroxyvitamin D
