# Supplementary material for: Lower Levels of Vitamin D Are Associated with an Increase in Insulin Resistance in Obese Brazilian Women
Source: Nutrients. 2021 Aug 27;13(9):2979. doi: 10.3390/nu13092979 (PMC8471993; doi:10.3390/nu13092979)
Supplement: Supplementary file 1 [file nutrients-13-02979-s001.zip › nutrients-1327582-supplementary.pdf]

Supplementary Materials: The following are available online at www.mdpi.com/xxx/s1, s2

Table S1. Clinical and biochemical characteristics of the population by obesity type.

Characteristics	All (n=103)	Obesity I (n=32)	Obesity II (n=40)	Obesity III (n=31)	p-value
**Age (years) - median (IQR)**	42 (34-52)	47 (37-55)	38 (34-51)	45 (33-53)	0.193
**Race - n (%)**					0.098
White	13 (13.8)	1 (3.2)	7 (19.4)	5 (18.5)	
Pardo	40 (42.6)	15 (48.4)	12 (33.3)	13 (48.1)	
Black	41 (43.6)	15 (48.4)	17 (47.2)	9 (33.3)	
**Waist circumference - median (IQR)**	109 (103-116)	103 (97-108)	111 (104-115)	116 (110-130)	<0.001
**BMI (kg/m^2)-median (IQR)**	37.5 (33.7-40.2)	32.7 (31.0-33.5)	37.5 (36.1-39.3)	44.3 (42.0-46.9)	<0.001
**Smoking (current) -n (%)**	2 (2.0)	0 (0.0)	2 (5.1)	0 (0.0)	0.986
**Smoking (past) -n (%)**	16 (17.8)	6 (22.2)	4 (11.1)	6 (22.2)	0.203
**Passive smoking -n (%)**	13 (13.3)	6 (19.4)	5 (13.2)	2 (6.9)	0.157
**Alcohol consumption (current)-n (%)**	33 (33.0)	10 (31.3)	11 (28.9)	12 (40.0)	0.475
**Alcohol consumption (past)-n (%)**	16 (17.8)	6 (22.2)	4 (11.1)	6 (22.2)	1
**Use of obesity medication -n (%)**	17 (53.1)	4 (57.1)	4 (44.4)	9 (56.3)	0.924
**Physical Activity -n (%)**	27(26.2)	10(31.3)	11(28.9)	6(20)	0.325
**Hypertension -n (%)**	59 (57.8)	18 (56.3)	19 (48.7)	22 (71.0)	0.245
**Diabetes -n (%)**	22 (21.4)	12 (37.5)	4 (10.0)	6 (19.4)	0.077
**Biochemistry parameters**					
**Cholesterol (mg/dL) -median (IQR)**	194 (167-224)	188 (165-216)	199 (156-231)	198 (172-214)	0.802
**Triglycerides (mg/dL)-median (IQR)**	125 (91-168)	120 (89-144)	127 (79-173)	135 (111-184)	0.165
**LDL (mg/dL) -median (IQR)**	124 (104-153)	126 (103-142)	118 (103-161)	124 (112-137)	0.979
**HDL (mg/dL) -median (IQR)**	41 (36-48)	43 (39-48)	41 (36-47)	41 (35-51)	0.881
**TSH (mUI/mL) -median (IQR)**	1.82 (1.3-2.9)	1.6 (1.2-2.4)	1.7 (1.3-2.8)	2.57 (1.7-3.4)	0.024
**CRP (mg/L) -median (IQR)**	4.70 (2.8-9.2)	3.65 (1.4-6.3)	5.62 (3.4-10.5)	5.7 (3.1-12.5)	0.040
**FPG (mg/dL)-median (IQR)**	96 (92-107)	95 (85-109)	95 (93-104)	98 (93-108)	0.276
**HbA1c (%) -median (IQR)**	6.0 (5.5-6.6)	6.3 (5.6-6.7)	5.7 (5.4-6.4)	6.0 (5.6-6.9)	0.063
**Insulin (mUI/mL) -median (IQR)**	15.1 (11.2-20.9)	12.9 (10.8-16.8)	19.3 (13.0-24.0)	17.3 (10.3-23.7)	0.006
**HOMA-IR -median (IQR)**	3.9 (2.8-5.4)	3.1 (2.8-4.2)	4.4 (3.2-5.7)	4.5 (2.9-6.4)	0.031
**25(OH)D (ng/mL) -median (IQR)**	22.8 (19.6-26.2)	22.2 (19.0-25.2)	23.6 (19.8-26.6)	22.6 (20.2-26.5)	0.841
**25(OH)D level -n (%)**					0.531
Deficiency	67 (65)	19 (54.4)	25 (65.5)	23 (74.2)	
Insufficient	27 (26.2)	10 (31.3)	11 (27.5)	6 (19.4)	
Normal	9 (8.7)	3 (9.4)	4 (10.0)	2 (6.5)	

Table note: Data represent no. (%), except for age and BMI, which is presented as median and interquartile range (IQR).All: variable obesity and was divided in Obesity I: BMI 30.0 – 34.9 kg/m2; Obesity II: BMI= 35.0 – 39.9 kg/m2; Obesity III: BMI= ≥40.0 kg/m2. Physical activity was defined as at least 30 minutes of activity at least 3 times a week. Continuous variables were compared between Obesity status using Kruskal- Wallis test. Diabetes and hypertension were self-reported and diagnosed. **Abbreviatures:** BMI=Body mass index; LDH= Low Density Lipoproteins; HDL: High Density Lipoprotein; TSH= Thyroid stimulating hormone; CRP= C reactive protein; FPG= Fasting plasma glucose; HbA1c =Hemoglobin glycated; 25(OH)D=25-hydroxyvitamin D.

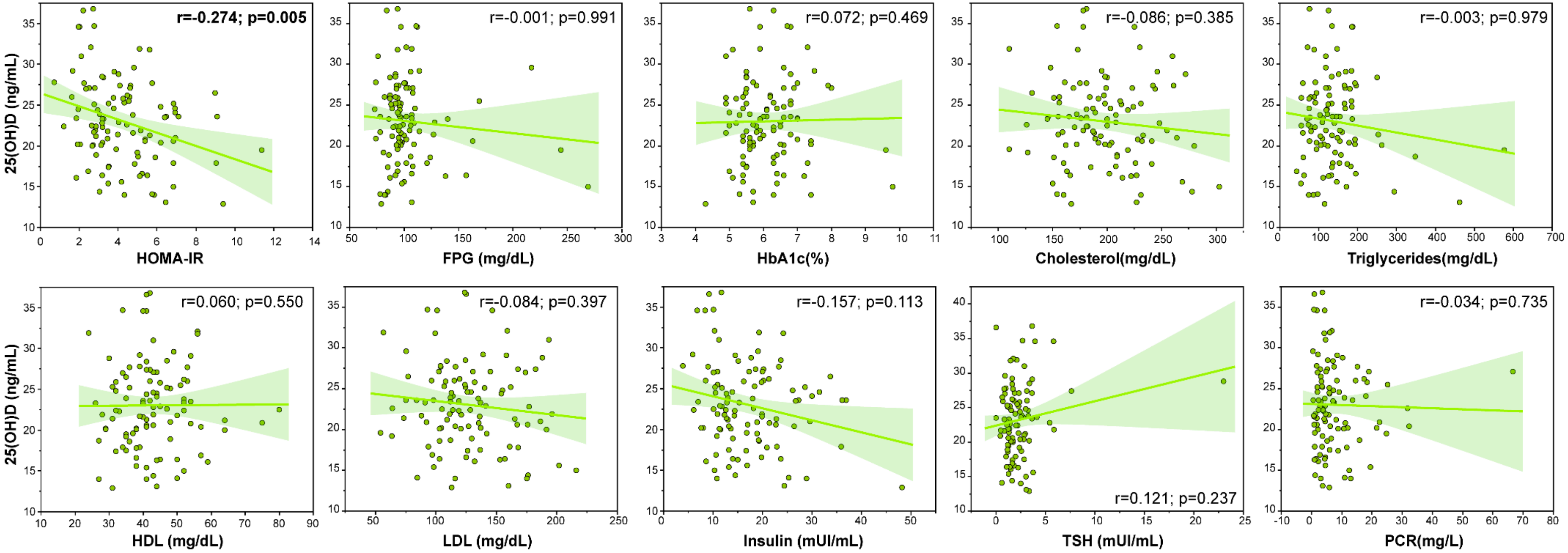

Figure S1. Spearman correlations analysis of the 25-hydroxyvitamin D [25(OH)D] and biochemical parameters in women with obesity. Correlation plot between 25-hydroxyvitamin D and all the biochemical parameters evaluated in the study.

Abbreviatures: FPG= Fasting plasma glucose; HbA1c =Hemoglobin glycated; HDL: High Density Lipoprotein; LDL= Low Density Lipoproteins; TSH= Thyroid stimulating hormone; C-RP= C-reactive protein; 25(OH)D=25-hydroxyvitamin D.
